# Effects of mesenchymal stromal cell-derived extracellular vesicles in acute respiratory distress syndrome (ARDS): Current understanding and future perspectives

**DOI:** 10.1002/JLB.3MR0321-545RR

**Published:** 2021-05-06

**Authors:** Yue Su, Haiyan Guo, Qinghua Liu

**Affiliations:** Department of Respiratory Medicine, Shanghai East Hospital, School of Medicine, Tongji University, Shanghai, P.R. China; Department of Paediatrics, The First Affiliated Hospital of Anhui Medical University, Hefei, Anhui, P.R. China; Department of Respiratory Medicine, Shanghai East Hospital, School of Medicine, Tongji University, Shanghai, P.R. China

**Keywords:** ARDS, COVID-19, extracellular vesicle, mesenchymal stromal cell

## Abstract

Acute respiratory distress syndrome (ARDS) is a devastating and life-threatening syndrome that results in high morbidity and mortality. Current pharmacologic treatments and mechanical ventilation have limited value in targeting the underlying pathophysiology of ARDS. Mesenchymal stromal cells (MSCs) have shown potent therapeutic advantages in experimental and clinical trials through direct cell-to-cell interaction and paracrine signaling. However, safety concerns and the indeterminate effects of MSCs have resulted in the investigation of MSC-derived extracellular vesicles (MSC-EVs) due to their low immunogenicity and tumorigenicity. Over the past decades, soluble proteins, microRNAs, and organelles packaged in EVs have been identified as efficacious molecules to orchestrate nearby immune responses, which attenuate acute lung injury by facilitating pulmonary epithelium repair, reducing acute inflammation, and restoring pulmonary vascular leakage. Even though MSC-EVs possess similar bio-functional effects to their parental cells, there remains existing barriers to employing this alternative from bench to bedside. Here, we summarize the current established research in respect of molecular mechanisms of MSC-EV effects in ARDS and highlight the future challenges of MSC-EVs for clinical application.

## Graphical Abstract

Describes the potential of Mesenchymal Stromal Cell-derived Extracellular Vesicles for the Treatment of Acute Respiratory Distress Syndrome

**Graphical Abstract:**
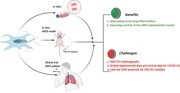

## INTRODUCTION

Acute respiratory distress syndrome (ARDS), as a hallmark of intensive care medicine, was first described more than 50 yr ago, although the definition has changed over time.^1^ To date, it is well recognized that ARDS is not a disease but a heterogeneous syndrome characterized by diffuse alveolar and endothelial damage, resulting in acute onset of widespread inflammation in the lungs.^2^ Over the last 50 yr, research into the epidemiologic features of ARDS has demonstrated that its morbidity and mortality show variability in geographic statistics in relation to local clinical conditions, clinicians’ recognition of ARDS, and methodologic differences, but a recent large observational study (LUNG SAFE) conducted across 50 countries has shown that the ARDS accounts for 10.4% of ICU admissions with mortality ranging from 35% to 46% in hospitals.^3^ Additionally, another study has estimated that annually ARDS affects more than 3 million patients worldwide and leads to 75,000 deaths in the United States.^4^ Nevertheless, low efficacy of the existing pharmacologic interventions of ARDS contributes to new potential therapeutic options being sought by investigators.

Multipotent mesenchymal stromal cells (MSCs) have been shown to possess the properties of immunomodulation and tissue repair in both experimental acute lung injury (ALI) and sepsis models through the secretion of several growth factors (TNF-α-stimulated gene-6), KGF (keratinocyte growth factor), prostaglandin E2, et al.), and anti-inflammatory cytokines (IL-10).^5–7^ Moreover, in an ex vivo model of perfused human lungs injured with live *E. coli* bacteria, MSCs were also capable of decreasing lung inflammation, clearing alveolar fluid, and enhancing alveolar macrophage phagocytosis.^8^ Despite the existence of data that have shown the benefit of MSCs for ARDS, a growing body of evidence is emerging querying the actual effects of MSCs in clinical practice in terms of the outcome of several completed randomized phase I/II clinical trials^9–12^ (Table 1). Although these early clinical trials have shown that MSCs did not induce prespecified infusion-related adverse events, there was no difference in 28 d mortality between the groups of MSCs and placebo, and the MSC group had higher mean scores of Acute Physiology and Chronic Health Evaluation III, which can provide initial risk stratification and risk estimates for critically ill patients, and higher scores correspond to more severe illness and a higher risk of death.^10,11^ Additionally, it has been proven that the efficacy of MSC engraftment and differentiation is limited^13^ and MSCs are short-lived cells, which are no longer viable after 24 h in the damaged lung by i.v. infusion.^14–16^ Of more concern is the fact that MSCs exhibit a high risk of carcinogenesis, and the possibility of tumorigenicity increases when MSCs are expanded in culture, whereas the long-term follow-up of MSC administration is absent in most preclinical experiments and clinical trials.^17,18^

**TABLE 1:** Clinical trials of mesenchymal stromal cells (MSCs) in acute respiratory distress syndrome (ARDS) (completed)

Clinical trial ID	study	Name of clinical trial	Phase	**MSC type**	**Dose**	**Frequency**	**Route**	**Patients Enrolled**	**Follow up**
NCT01902082	Zheng et al.^9^	Adipose-derived mesenchymal stem cells in acute respiratory distress syndrome	Phase 1	AD-MSCs	1 × 10^6^/kg	once	i.v.	20(10/10)	28 days
NCT01775774	Wilson et al.^10^	Human mesenchymal stromal cells for acute respiratory distress syndrome (START)	Phase 1	BM-MSC	1,5,10 × 10^6^/kg	once	i.v.	9(3/3/3)	12 months
NCT02097641	Matthay et al.^11^	START	Phase2a	BM-MSC	1 × 10^7^/kg	once	i.v.	60(40/20)	12 months
NCT02611609	Bellinga et al.^12^	A phase 1/2 study to assess multistem therapy in acute respiratory distress syndrome (MUST-ARDS)	Phase 1/2	MultiStem	300/900million	once	i.v.	30(10/10/10)	12 months
NCT02095444	Chen et al.^77^	Using human menstrual blood cells to treat acute lung injury caused by H7N9 bird flu virus infection	Phase 1/2	MB-MSC	1∼10 × 10^7^ cells/kg	4 times	i.v.	20	5 years

Recently, attention has been drawn to MSC-derived extracellular vesicles (MSC-EVs) as a new frontier in the cell-free treatment regime for ARDS. There are increasing data to suggest that the properties of MSC-EVs are similar to their parental cells in anti-inflammation and tissue homeostasis in damaged cells or diseased organs. Furthermore, the characteristics of no risk of tumorigenicity, and a lower possibility of immunologic rejection and self-replication, make them a promising candidate for the treatment of ARDS.^19^

## EVS: DEFINITION AND BIOGENESIS

**FIGURE 1:**
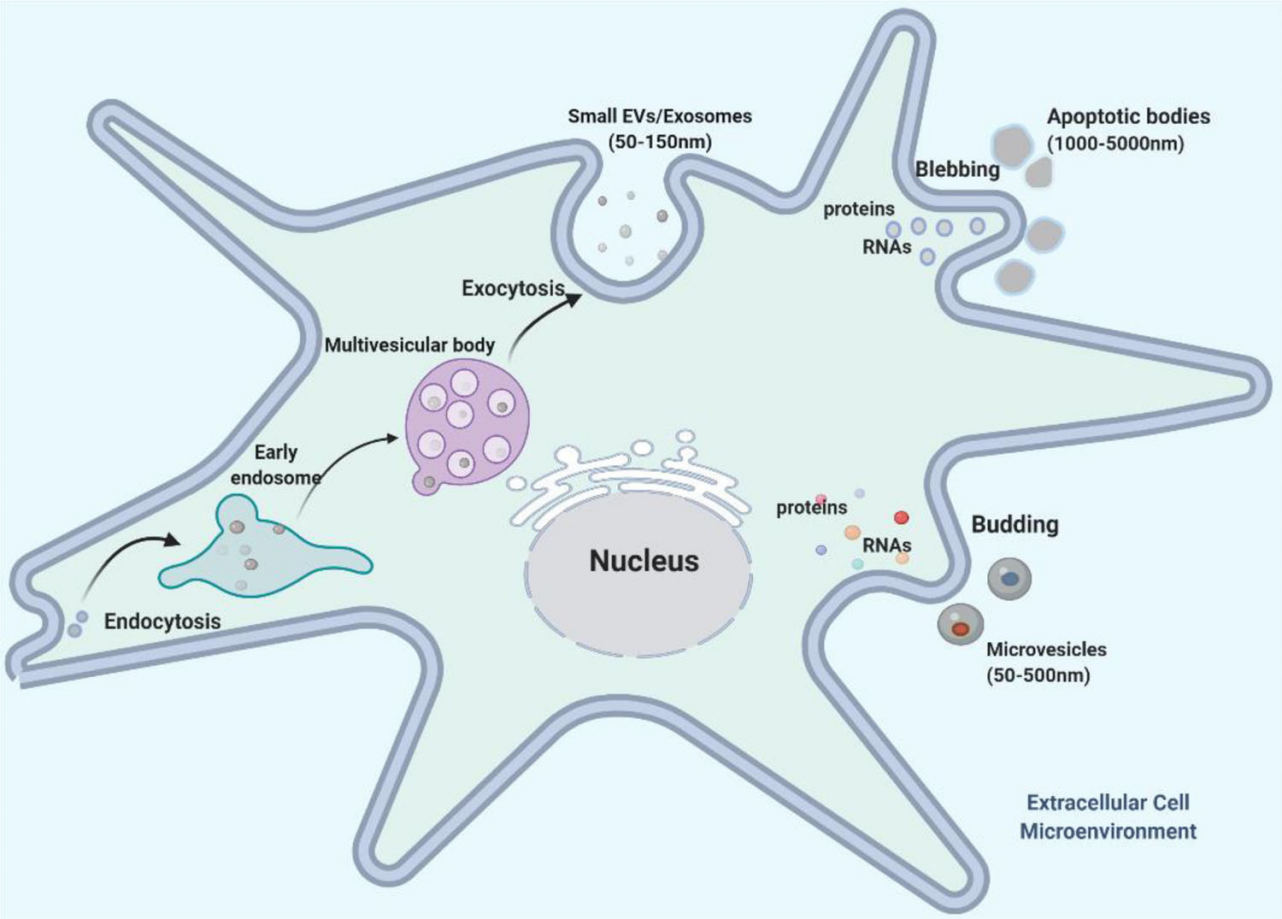
**Mesenchymal stromal cell extracellular vesicle (MSC-EV) biogenesis and contents.** MSC-EV subtypes (exosomes, microvesicles, and apoptotic bodies) present three distinct molecular biogenesis pathways. Exosomes are nano-sized (50–150 nm) bioparticles released upon fusion of multivesicular bodies (MVB) with the plasma membrane, carrying a conserved set of proteins (heat shock protein [HSP70/90], CD9/63/87). Microvesicles are 100–1000 nm in size shed from cell membrane through direct outward budding. Apoptotic bodies (ApoBDs) are defined as 1000–5000 nm in diameter formed during plasma membrane blebbing in late stage of apoptosis

EVs are lipid bilayer-surrounded particles, comprising various subpopulations of released cells, which participate in multiple physiologic and pathologic activities to facilitate intercellular communications and to change the biologic components of recipient cells.^20^ Based on the International Society for Extracellular Vesicles (ISEV) proposed guidelines of minimal information for studies of extracellular vesicles 2018, researchers are encouraged to consider using operational terms of EV subpopulations that refer to (i) physical characteristics of EVs, including size and density; (ii) biochemical composition, such as Annexin A5-stained EVs; and (iii) descriptions of conditions or cell origin, such as hypoxic EVs. However, in the current published studies, most researchers use the terms “exosomes” (endosome, 50–150 nm), “microvesicles” (plasma membrane, 50–500 nm), and “apoptotic bodies” (1000–5000 nm) to divide EVs into three main categories based on size and composition (Fig. 1). Mounting evidence has shown that EVs display a commonly conserved set of chaperones (heat shock protein70 [HSP70], HSP90), membrane organizers (CD9, CD63, CD81), and cell-type-specific protein (MHC-I).^21^ Nevertheless, in regard to different types of cellular sources and microenvironments, exosomes and microvesicles have presented specific contents of their cargos (varying from genetic material to lipids and proteins) that are associated with different targeted cells and various physiologic functions.

In essence, exosomes and microvesicles represent two distinct molecular mechanisms of EV biogenesis and shedding. Briefly, exosomes are derived from endosomes or multivesicular bodies (MVBs) generated by the inward budding of the endosomal system. After the process of endocytosis, the intraluminal vesicles may fuse either with lysosomes or with plasma membrane, the results of which are called exosomes. Somewhat differently, during the maturation of microvesicles, they integrated with plasma membrane through outward budding and are subsequently released into extracellular space to deliver biologically relevant information to recipient cells.^22^

## MECHANISM DISSECTION OF MSC-EV EFFECTS IN ARDS

**FIGURE 2:**
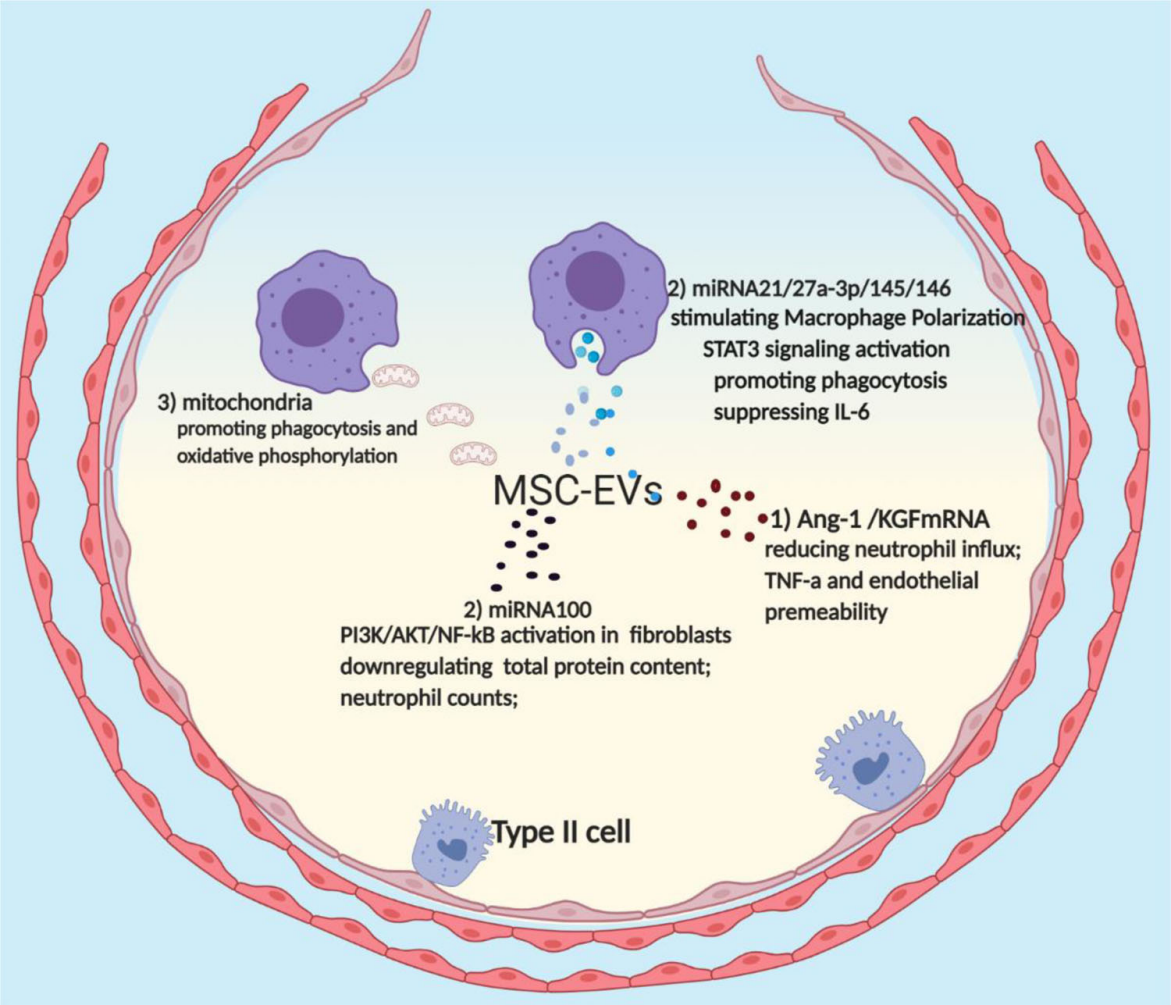
**Therapeutic effects of mesenchymal stromal cell extracellular vesicles (MSC-EVs) in acute respiratory distress syndrome (ARDS).** mRNA, microRNA (miRNA), and mitochondria packaged in MSC-EVs have shown great immunosuppressive and reparative effects in ARDS. (1) mRNA: MSC-EVs deliver mRNA fragments of keratinocyte growth factor (KGF) and angiopoietin 1 (Ang-1) have shown great therapeutic effects in decreasing neutrophil infiltration, increasing anti-inflammatory cytokine production, down-regulating proinflammatory cytokine secretion, total protein, and vascular endothelial permeability. (2) miRNA: MSC-EVs are capable of transferring miRNA21, miRNA27a-3p, miR145, and miR146 to alveolar macrophages, contributing to M2 macrophage polarization, STAT3 signaling activation, macrophage phagocytosis promotion, and down-regulate IL-6 secretion. Furthermore, miR100 can be delivered to fibroblast cells by MSC-EVs, resulting in PI3K/protein kinase B (AKT)/NF-kB signaling activation, down-regulation of total protein content, neutrophil counts, and proinflammatory levels. (3) Mitochondria: MSCs donate their mitochondria to macrophages through nanotubes or EVs, facilitating oxidative phosphorylation and phagocytosis of macrophages

MSC-EVs have been demonstrated to have therapeutic benefits for ARDS and severe pneumonia in preclinical studies. The main bio-functionalized benefits of MSC-EVs have been presented in the aspect of attenuating acute inflammation, promoting alveolar epithelial regeneration, and enhancing pulmonary endothelial repair.^23^ In this context, accumulating evidence aiming at dissecting molecular mechanisms has shown that MSC-EVs as a shuttle of bio-active messengers is capable of wrapping mRNAs, proteins, microRNA (miRNAs), and mitochondria to modulate immune responses and to repair widespread lung damage in the exudative, proliferative, and fibrotic phases of ARDS, which assist in decreasing proinflammatory cytokine production and improving alveolar fluid clearance^12^ (Fig. 2).

### Role of mRNAs in MSC-EVs in ARDS

The mRNA is a single-stranded molecule that carries genetic information copied from DNA for protein synthesis.^24^ Batagov and colleagues showed that most exosomal mRNAs that are enriched with specific 3′-untranslated regions that may present as a competing RNA in targeted cells to modulate the physiologic processes.^25^ MSC-EVs packaged mRNA fragments of KGF and angiopoietin 1 (Ang-1) have shown great therapeutic effects in restoring ALI injury.

#### KGF mRNA

KGF is a human mitogen secreted from mesenchymal cells, which plays a key role in enhancing alveolar type epithelial II cell proliferation and DNA repair, and inhibiting oxidant-induced epithelial cell permeability.^26,27^ Zhu and coworkers have demonstrated that MSC-microvesicles (MSC-MVs) are able to relieve the severity of lung injury in the mouse model of *E. coli* endotoxin-induced ALI.^28^ More specifically, compared to a placebo group, MSC-MVs exert a positive impact on down-regulating the levels of neutrophil infiltration and macrophage inflammatory protein-2 by 73% and 49%, respectively, reducing alveolar lung fluid by 43%, and also decreasing total protein level in bronchoalveolar lavage fluid (BALF) by 35%. Furthermore, suppressing KGF mRNA expression in MSC-MVs by KGF siRNA is able to reverse the beneficial effects of MSC-EVs, which suggests the potent therapeutic effects of MSC-MVs are partially derived from the delivery of KGF mRNA to recipient cells.

#### Ang-1 mRNA

Ang-1 is an oligomeric secreted glycoprotein that is required for attenuating plasma permeability, relieving vascular inflammation, preventing endothelial injury, and maintaining vascular stabilization.^29^ Previous studies demonstrated that Ang-1 gene-transduced MSCs show a greater improvement in restoring acute alveolar injury and reducing pulmonary vascular endothelial permeability than MSCs or Ang-1 alone.^30,31^ Recently, Tang et al. have shown that down-regulating Ang-1 mRNA by a lentivirus vector carrying Ang-1 short hairpin RNA in MSC-MVs fails to decrease the production of proinflammatory cytokine (TNF-α) and to raise IL-10 production in macrophages.^32^ Moreover, Ang-1 mRNA deficient MVs lose the effect of protecting the integrity of LPS-induced microvascular endothelial cells and of decreasing neutrophil influx. These robust data elucidate that MSCs can transfer Ang-1 mRNA to endotheliocytes and macrophages to regulate wound repair and immune responses.

Even the results have shown ALI can be restored by MSC-EV wrapped KGF and Ang-1 mRNA, the standardization, quantification, and characterization of MSC-EVs are absent in these two studies. Moreover, the potential targets and molecular mechanisms for KGF and Ang-1 mRNA in the alveoli remain uninvestigated.

### Effects of MSC-EVs transferred miRNAs in ARDS

The miRNAs are a class of small noncoding RNAs that are transcribed from DNA sequences, normally 22 nucleotides in length,^33^ which exert a critical impact on various biologic activities. Mechanically, miRNAs secreted into EVs or fluids are able to interact with targeted mRNAs, thereby suppressing protein translation. However, in the context of several specific microenvironments, miRNAs also promote or modulate mRNA translation. Accumulating evidence has shown that MSC-EVs transfer miR-21p, miR-27p-3a, miR30b-3p, miR100, miR145, and miR146 to injured or inflamed lung tissue for regulating their biogenesis and homeostasis.

#### miR21

MiR21 is one of most investigated and responsive miRNAs in the context of distinct pathologic processes, including acute or chronic lung inflammation, diabetic complications, and myocardial ischemia. It has been demonstrated that miR21 is able to down-regulate STAT3 signaling to suppress LPS-induced ALI by decreasing the IL-6 secretion in murine RAW264.7 cells.^34^ In what could be another mechanism for miR21 biologic function in lung disease, miR21 facilitates the protective effect of long noncoding RNA NF-κB interacting LncRNA by inhibiting NF-κB and JNK intracellular signaling pathways of medical research council cell strain 5 cells (human fibroblast cell line) in the experimental model of infantile pneumonia.^35^ Recently, Li and coworkers have stated that MSC exosomes transport anti-apoptotic miR21-5p to alveolar macrophages, which leads to the macrophages shifting toward a predominantly M2 phenotype and also to the down-regulation of proinflammatory cytokine secretion by inhibiting intrinsic or extrinsic pathways through phosphatase and tensin homolog or programmed cell death 4.^36^

#### miR27

Over the past a few years, miR27a/b has been shown to have an important role in neovascularization and granulocyte differentiation, which enhances capillary sprouting and endothelial tip fate through inhibiting Notch signaling, the Delta-like ligand 4 and sprouty-2.^37,38^ Research has shown that miR27 is involved in attenuating acute liver inflammation and oxidative stress through decreasing the levels of TNF-α and IL-6, and elevating the production of superoxide dismutase and glutathione peroxidase via miR27-TAB3-NF-κB intracellular signaling pathways in the mouse model of sepsis.^39^ More recently, Xu et al. have conducted an innovative investigation, which shows that miR27a-3p wrapped in MSC-EVs are taken up by alveolar macrophages and subsequently decrease their NF-κB expression. Additionally, lentiviral transduction of miR27a-3p with anti-miR27a-3p or knockdown of miR27a-3p mitigates the benefits of MSC-EVs in ALI and M2 polarization.^40^

#### miR30

It has been illuminated that the miR30 family (including miR30a, b, c, d, e), as tumor inhibitors, possesses the ability to suppress epithelial-mesenchymal transition in cancer cells, and overexpression of miR30s is responsible for reducing pulmonary vascular hyperpermeability to postpone cancer proliferation and metastasis through targeting S-phase kinase-associated protein 2 directly.^41^ Furthermore, data from miRNA microarrays from the lung tissue of idiopathic pulmonary fibrosis patients have shown that several member expressions of the miR30 family, including miR30b, c, d, are significantly down-regulated, which reveals that the miR30 family is associated with many biologic processes in the lungs.^42^ Remarkably, recent evidence from Yi and colleagues has validated that miR30b-3p packaged in MSC exosomes is able to enhance the proliferation and to suppress the apoptosis of MLE-12 cells (mouse alveolar type II epithelial cell line) in the presence of LPS through inhibiting serum amyloid A 3 (SAA3) expression. Moreover, PKH26-labelled miR30b-3p exosomes can be delivered into injured lung tissue and miR30b-3p overexpression in exosomes protects the structure of alveolar cells, decreases the volume of alveolar edema, and down-regulates proinflammatory soluble factors (SAA3, IL-1β, TNF-α, and IL-6) in the BALF of the LPS-induced ALI mice model.^43^

#### miR100

Most studies into miR100 in lung diseases concern its antitumor effects and its role as a potential molecular prognostic marker in nonsmall lung cancer.^44,45^ Recently, miR100 has been demonstrated to modulate LPS-induced apoptosis and autophagy of WI-38 cells (normal human fibroblast cell line) through activating PI3K/protein kinase B/NF-κ-light-chain-enhancer of activated B cells (PI3K/AKT/NF-κB) intracellular pathway in the experimental model of acute pneumonia.^46^ More importantly, Chen et al. have indicated that miR100 similarly accounts for the therapeutic benefits of Wharton's jelly MSC-EVs partially by attenuating the bleomycin (BLM)-induced apoptosis and inflammation of the rat type II alveolar epithelial cell line (L2 cells). Moreover, in this study, MHY1485 as an autophagy inhibitor, down-regulates the autophagic effect of MSC-EVs in BLM-treated L2 cells coupled with a decreased anti-inflammatory effect, which suggests the miR100-MSC-EVs delivery mitigates BLM-induced ALI through enhancing autophagy. Additionally, miR100 overexpressed MSC-EVs attenuate the total protein content, neutrophil counts, and the proinflammatory levels in the BALF, and decrease cell apoptosis in the BLM-induced ALI rat model.^47^

#### miR145

MiR145 is engaged in normal blood cell development and appears to play a fundamental role in the biogenesis and division of megakaryocytes.^48^ Regarding lung diseases, miR145 has been reported to contribute to the pathogenesis of hypoxia-induced pulmonary artery hypertension, and miR145 deficiency has also modulated myofibroblast differentiation to protect BLM-treated lung fibrosis.^49,50^ Nevertheless, the expression of miR145 has been down-regulated in the exosomes of septic patients’ blood samples and lung tissue from septic mice, and miR145 attenuates LPS-induced septic lung injury and acute lung inflammation through targeting TGF beta receptor II and inactivating TGF-β/Smad signaling.^51^ Similarly, investigation into the MSC-EV effect on ALI by Hao has reported that miR145 is capable of transferring into murine macrophages to promote phagocytosis and to decrease *E. coli* bacterial load in acute pneumonia through increasing leukotriene B4. Further experiments have also shown that miR145 regulates multidrug resistance protein 1, which is a member of the ATP-binding cassette transporters and also elevates the levels of leukotriene A4 hydrolase and matrix metallopeptidase 9.^52^

#### miR146

The miR146 family, consisting of miR146a and miR146b, has been shown to be a well-documented anti-inflammatory miRNA and a negative mediator of inflammatory gene expression in human alveolar epithelial cells and airway smooth muscle.^53–55^ Moreover, the plasmacytoma variant translocation gene, as an antagonist of miR146, has been reported to down-regulate miR146 expression in the sputum of chronic obstructive pulmonary disease patients and smokers.^56^ Additionally, up-regulating miR146 protects *E. coli*-induced early sepsis in mice through increasing GSKJ4 expression and inhibiting NF-kB p65 signaling pathway.^57^

In a cecal ligation and punctual-induced sepsis model, Wang et al. were the first to demonstrate that IL-1β-primed MSCs promote the mouse survival rate via reducing bacterial burden, decreasing proinflammatory cytokines, and elevating anti-inflammatory cytokines. Subsequently, they have investigated that MSC transferred miR146 contributes to macrophage polarization to M2 phenotype, and suppressing miR146 expression in MSC exosomes could partially negate the immunomodulatory effects of MSC-EVs.^58^

These promising results have suggested that MSC-EV-delivered miRNAs play a dominant role in restoring ARDS. Nevertheless, most experiments mentioned earlier were only conducted in murine cells or human cell lines, and no clinical samples or clinical biologic variables are used or considered in these studies. Furthermore, these studies did not present the bio-distribution of MSC-EVs in the bodies of the experimental mice and the efficacy of MSC-EVs was also undetected in comparison with MSCs alone.

### Benefits of MSC-EV packaged mitochondria in ARDS

Mitochondria, referred to as the powerhouse of the cell, are believed to exert a vital impact on the pathologic processes of the illness by regulating cell metabolism and homeostasis. Optimal performance of cellular mitochondria relies on the sophisticated and dynamic system that provides a complete electron transport chain (ETC), an efficient citric acid cycle, and intact mitochondria constituents. Conversely, damaged ETC, excessive superoxide-derived reactive oxygen species production, unbalanced cytosolic and mitochondrial calcium levels, and morphologic changes (mitochondria leak and uncoupling) contribute to mitochondrial dysfunction and cell injury.^59,60^ Of note, mitochondrial bio-energetic failure also plays a key role in the pathogenesis of ARDS.^61^ Ever-increasing evidence has suggested mitochondrial dyshomeostasis is implicated in ARDS development and pathophysiology.^62^

A preclinical study of ARDS has revealed that significant ATP decline and low arterial oxygenation have been shown in the LPS-treated mouse model, which are considered to be associated with prolyl hydroxylases and hypoxia-inducible factor.^63^ The preliminary data from Ten et al. have demonstrated that ADP-phosphorylating respiration in mitochondria isolated from mouse injured lungs that were treated with LPS is lower than the control group.^64^

To the best of our knowledge, Spees and coworkers have initially proved that MSCs rescue the aerobic respiration of mitochondria DNA (mtDNA) depleted-A549 cells (alveolar basal epithelial cell line). Moreover, single nucleotide polymorphism analysis has found that the recipient A549 cells contain the mtDNA, which belongs to the donor cells.^65^ Furthermore, in order to identify the potential mechanisms and the extent of protective effects of MSC transferring mitochondria to alveolar epithelial cells in ALI, the study from Islam et al. was the first to report that homing MSCs secrete microvesicles and generate mitochondria transporting nanotubes to the alveolar epithelial cells to elevate ATP production in the inflamed lung and to protect against ALI. Additionally, connexion 43 (also known as gap junction alpha-1)-expressed in bone marrow-derived MSCs is responsible for MSCs generating gap junctional channels to attach to the alveoli.^66^

More recently, the underlying mechanisms of how mitochondria are delivered from MSCs to immune cells and modulate immune responses have been dissected by Jackson and colleagues, who have illustrated that extensive mitochondria are delivered to macrophages partially through tunneling nanotube-like structures, and these transferred mitochondria result in an enhancement of macrophage phagocytosis.^67^ Moreover, they have also established that depleting alveolar macrophages by liposomal clodronate leads to complete abrogation of MSC therapeutic effects in the mouse model of *E. coli* pneumonia. In order to determine whether MSC-EVs could package mitochondria to alveolar macrophages and modulate macrophage metabolism in a clinically relevant model, Morrison et al. have reported that in an ARDS patients’ BALF treated-lung injury model, adoptive transfer of mitochondria from MSCs to macrophages via the CD44 negative EV carrier ameliorates acute lung inflammation by elevating oxidative phosphorylation and the phagocytic ability of macrophages.^68^

Nonetheless, the therapeutic benefits of MSCs’ mitochondria transfer in ARDS still remain ambiguous. In an ALI model induced by hypercapnic acidosis (HCA), which is associated with low tidal volume ventilation, HCA-induced mitochondria dysfunction impairs the capacity of MSCs to promote epithelial wound closure, suggesting that MSCs lose their therapeutic effects of mitochondria transfer to epithelial cells in the HCA microenvironment.^69^

## CLINICAL POSSIBILITY OF MSC-EVS FOR COVID-19

A novel human coronavirus, named SARS-CoV-2, was found in some severe pneumonia cases in early December 2019, and within several months had caused a pandemic of respiratory illness termed COVID-19.^70^ This epidemic is now spreading in over 200 countries and territories around the world through human-to-human transmission. Recently, a global literature survey has revealed that of hospitalized COVID-19 patients, approximately one-third (33%) develop ARDS, one-fourth (26%) need to be transferred into ICU, and one-sixth (16%) cases die in the hospital.^71^ Previous studies, including clinical trials, have shown that MSCs or MSC-EVs are beneficial to H1N1, H5N1, H9N2, or H7N9-induced ALI or pneumonia, presenting a potent effect on decreasing proinflammatory cytokine secretion and suppressing immune cell recruitment in the lungs^72–77^ (Table 2). Due to there being no precise and specific antiviral medicine for this emerging illness, MSCs were applied in several critically ill COVID-19 patients, contributing to the decline of plasma C reactive protein (CRP), aspartic aminotransferase, creatine kinase activity, and myoglobin. Furthermore, combined with symptomatic and supportive treatment, chest CT manifestation and SARS-CoV-2 nucleic acid detection improved markedly or returned to negative after 2 wk.^78–81^ Although the positive results mentioned earlier supported the administration of MSC-EVs as a safe and efficient alternative for critically ill COVID-19 patients, the un-rigorous experimental design and small sample size of these studies contributed to these unimpressive and suspect data. Especially, only one clinical trial randomly divided subjects into MSC-treatment group and standard treatment group, and no clinical trials have tested the MSC viability before infusion.

**TABLE 2:** Completed preclinical and clinical trials of mesenchymal stromal cells (MSCs) or MSC-EV treatments in Influenza-associated acute respiratory distress syndrome (ARDS)

	Model disease	Study	MSC			Dose	Frequency	Route	Results	Outcome
Preclinical trial	H1N1	Darwish et al.^16^	BM-MSC			5 × 10^5^ cells	Once	i.v.	Failed to improve survival, decrease pulmonary inflammation/inflammatory cell counts or prevent acute lung injury (ALI) in influenza virus-infected mice	MSCs failed to improve survival rate in experimental severe influenza
	H1N1	Gotts et al.^76^	BM-MSC			5 × 10^5^ cells	Once	i.v.	Biologically active MSCs were delivered to the lungs and reduce lung viral load, but MSCs did not improve influenza-induced lung injury	Influenza caused lung injury unresponsive to MSCs in mice
	H1N1	Khatri et al.^75^	BM-MSC-EV			79 ± 1 μg/100 μl EVs	Once	i.v.	Inhibit HA activity of influenza viruses, virus replication and virus-induced apoptosis, inflammatory cytokines and ALI	MSC-EVs alleviated influenza virus-induced lung lesions in pigs
	H5N1	Chan et al.^73^	BM-MSC			5 × 10^5^ cells	Once	i.v.	Reduced AFC, proinflammatory cytokine secretion and enhances APP in human alveolar epithelial cells, sodium and chloride transporter protein expression, restore lung injury through angiopoietin 1 (Ang-1) and keratinocyte growth factor (KGF) production	MSCs reduced H5N1-associated ALI injury in mice
	H5N1	Loy et al.^74^	UC-MSC			5 × 10^5^ cells	Once	i.v.	Reduced H5N1-induced down-regulation of ion transporters, lung proinflammatory responses; restore H5N1-impaired AFC and APP partially through Ang-1 and HGF secretion	UC-MSCs were effective in restoring H5N1-associated ALI than BM-MSCs
	H9N2	Li et al.^72^	BM-MSC	1 × 10^5^ cells			Once	i.v.	Decreased dry weight ratios, airspace inflammation, ERK and JNK, and expression the chemokine concentration (GM-CSF, MCP-1, KC, MIP-1α, and MIG), PaCO2; improve survival rate, lung histopathology, PaO2, SaO2, PH	MSCs prevented H9N2 avian influenza virus-induced ALI in mice
Clinical trial	H7N9	Chen et al.^77^	MB-MSCs			1 ×10^6^/kg	4 times	i.v.	Down-regulated the productions of PCT, plasma C reactive protein (CRP), serum creatinine, creatine kinase (CK), prothrombin time (PT), and D-dimer	MSCs improved the survival rate of H7N9-induced ARDS

Recently, a prospective nonrandomized open-label cohort study was conducted to evaluate the safety and efficacy of allogeneic bone marrow MSC-derived exosomes (ExoFlo) in severe COVID19 patients who suffered from moderate to severe ARDS.^82^ To this end, 24 patients were i.v. administrated 15 ml ExoFlo (single dose) that was added into 100 ml normal saline solution. In the 72 h of post-treatment, no adverse events were observed related to the administration. After a 2 wk observation, 17 patients (71%) recovered, 3 patients (13%) remained severely ill, and 4 patients (16%) died. The laboratory results of the patients have shown that the levels of CRP, ferritin, and D-dimer, and neutrophil count decreased, whereas the average CD3+, CD4+, and CD8+ lymphocyte count increased. However, the paper did not mention how the ExoFlo was developed and manufactured, and there appears to be no difference in mortality between this cohort and that of the global survey (Table 3). To date, even though several clinical trials are ongoing to determine the effects of MSC-EVs for COVID19, the International Society of Cell and Gene Therapy and ISEV strongly urge that the decision to apply of MSC-EVs for COVID should be taken carefully due to the limited preclinical and clinical data, and they also suggest any use of EVs should be carefully evaluated through rational clinical trial design.^83^

**TABLE 3:** Clinical effects of mesenchymal stromal cells (MSCs) or MSC-EVs in COVID19

Name of the clinical trial	Study	Dose	Frequency	Route	Patient enrolled	Survival rate	Laboratory outcomes	Image outcomes (X ray/CT)
Treatment of severe COVID-19 with human umbilical cord mesenchymal stem cells (randomized)	Shu et al.^79^	2 × 106 cells/kg	Once	i.v	41(MSC-12/Placebo-29)	100%	↓ Plasma C reactive protein (CRP), IL-6; ↑oxygenation index, lymphocyte count	CT scores, the number of lobes involved, GGO, and consolidation better than control group
Human umbilical cord-derived mesenchymal stem cell therapy in patients with COVID-19: a phase 1 clinical trial (nonrandomized)	Meng et al.^80^	3 × 107 cells	three cycles	i.v	18(MSC-9/Placebo-9)	100%	↓ IL-6; ↑PaO2/FiO2, lymphocyte count	N/A
Adipose-derived mesenchymal stromal cells for the treatment of patients with severe SARS-CoV-2 pneumonia requiring mechanical ventilation (nonrandomized)	Sanchez-Guijo et al.^81^	0.98 × 106 cells/kg	1/2/3 cycles	i.v	13	85%	↓CRP, IL-6, LDH, D-dimer; ferritin; ↑lymphocyte count, CD4+/CD8+ T cell	Radiologic improvement in 40% of evaluable patients
Exosomes Derived from bone marrow mesenchymal stem cells as treatment for severe COVID-19 (nonrandomized)	Sengupta et al.^82^	15 ml	Once	i.v	24	83%	↓CRP, D-dimer, ferritin; ↑neutrophil count, CD3+, CD4+, and CD8+ lymphocyte counts	N/A

## BARRIERS IN MSC-EV APPLICATION IN ARDS

Despite multiple studies that have shown the promising evidence that MSC-EVs are beneficial for ARDS, there remain several issues that need to be settled before it can be applied as an off the shelf alternative in clinics.

MSCs are a cluster of multilineage potential cells that can be derived from distinct tissues,^84^ such as bone marrow, placenta, adipose tissue, among others. A large body of evidence has shown that MSCs present tissue-to-tissue functional variation, and even the same source of MSCs still exhibit different proliferation capacities as well as immunosuppressive ability.^85^ EVs isolated from various sources of MSCs are also not a homogenous system,^86^ and the differential effects of the EVs from young and aging MSCs in ALI have been established.^87^ The young MSC-EVs have shown similar therapeutic effects to those of their parental cells to alter macrophage phenotypes and to reduce monocyte recruitment, whereas aging MSC-EVs fail to shift macrophage polarization to an anti-inflammatory phenotype. Compared to the young EVs, the aging MSC-EVs have also presented a poorer internalization into macrophages and a lower content of miRNAs that are responsible for the protective benefits. Furthermore, systematic administration or local injection (intranasal or intratracheal) of MSCs exhibits differential impacts on ALI/ARDS preclinical models.^88^ To the best of our knowledge, no publications have been established to compare the different effects and bio-distribution of MSC-EVs through i.v. or intratracheal administration in ARDS. In this regard, optimizing our understanding of EV resources and delivery routes is required in the future.

ARDS is a pathogenic heterogeneous syndrome that contributes to various treatment responses and clinical outcomes in patients, for which to date the mortality remains high in ICU.^3^ In order to untangle the clinical and pathologic complexity and investigate tailored treatments for homogenous groups of ARDS patients, Calfee and colleagues initially identified the subphenotypes of ARDS, comprising a hypo-inflammatory subgroup and a hyper-inflammatory subgroup.^89^ Data from reanalysis of the clinical trials (ARMA, ALVEOLI, and HARP-2) have shown that approximately 30% of ARDS cases present higher levels of serum proinflammatory biomarkers, fewer ventilator-free days, and higher mortality than the hypo-inflammatory group.^90^ Even though clinical trials have not shown prominent positive effects in ARDS patients, the MSC-EV effects in the patients with ARDS subphenotypes remain uninvestigated.

Previous evidence from animal work has revealed that MSC-EVs may exhibit their promising effects at the dose of 1 × 10^8^ to 1 × 10^10^ nanoparticles,^52,91^ which suggests large quantities of MSC are needed for the extraction. More crucially, most laboratories across the globe use their in-house isolation protocols and characterization methodologies,^83^ which gives rise to ambiguities and inconsistencies when evaluating the promising results. Thus, developing good manufacturing practice protocols to scale up EV secretion, and establishing harmonized criteria for the standardization of EV production are urgently needed.

## CONCLUSION

The high morbidity and mortality of ARDS threaten millions of people worldwide, especially in this period of the COVID-19 pandemic. Currently, there remains no effective pharmacologic treatment for ARDS, and we are in a race to develop efficient strategies against this invisible enemy. Even though several translational issues need to be solved before MSC-EVs are applied in clinical work, this cell-free product still holds promise in reducing dysregulated acute immune responses, enhancing alveolar epithelial homeostasis, decreasing microvascular permeability, and preventing pulmonary fibrosis. Dissecting the underlying mechanisms, designing tailor-made regimens, and setting out harmonized manufacturing protocols are required for accelerating this alternative from bench to bedside.

## AUTHORSHIP

Y.S. wrote the manuscript; H.G. made the tables and drew the figure; and Q.L. supervised and checked the syntax of this article. All authors read and approved the final version of the manuscript for submitting to the *Journal of Leukocyte Biology*.

## DISCLOSURES

The authors declare no conflicts of interest.

## Abbreviations

AKT: protein kinase B
ALI: acute lung injury
Ang-1: angiopoietin 1
ARDS: acute respiratory distress syndrome
BALF: bronchoalveolar lavage fluid
BLM: bleomycin
CRP: plasma C reactive protein
ETC: electron transport chain
HCA: hypercapnic acidosis
HSP: heat shock protein
ISEV: International Society for Extracellular Vesicles
KGF: keratinocyte growth factor
miRNA: microRNA
MSC: mesenchymal stromal cell
MSC-EVs: MSC-derived extracellular vesicles
MSC-MVs: MSC-microvesicles
mtDNA: mitochondria DNA
MVBs: multivesicular bodies
SAA: serum amyloid A
